# Tissue dynamics spectroscopic imaging: functional imaging of heterogeneous cancer tissue

**DOI:** 10.1117/1.JBO.25.9.096006

**Published:** 2020-09-22

**Authors:** Zhe Li, Bihe Hu, Guang Li, Sharon E. Fox, Shadia I. Jalal, John Turek, J. Quincy Brown, David D. Nolte

**Affiliations:** a Purdue University, Department of Physics and Astronomy, West Lafayette, Indiana, United States; b Tulane University, Department of Biomedical Engineering, New Orleans, Louisiana, United States; c LSU Health Sciences Center, Department of Pathology, New Orleans, Louisiana, United States; d Indiana University School of Medicine, Department of Medicine, Indianapolis, Indiana, United States; e Purdue University, Department of Basic Medical Sciences, West Lafayette, Indiana, United States

**Keywords:** tissue-dynamics imaging, tumor spatial heterogeneity, functional imaging, deep optical imaging

## Abstract

**Significance**: Tumor heterogeneity poses a challenge for the chemotherapeutic treatment of cancer. Tissue dynamics spectroscopy captures dynamic contrast and can capture the response of living tissue to applied therapeutics, but the current analysis averages over the complicated spatial response of living biopsy samples.

**Aim**: To develop tissue dynamics spectroscopic imaging (TDSI) to map the heterogeneous spatial response of tumor tissue to anticancer drugs.

**Approach**: TDSI is applied to tumor spheroids grown from cell lines and to *ex vivo* living esophageal biopsy samples. Doppler fluctuation spectroscopy is performed on a voxel basis to extract spatial maps of biodynamic biomarkers. Functional images and bivariate spatial maps are produced using a bivariate color merge to represent the spatial distribution of pairs of signed drug-response biodynamic biomarkers.

**Results**: We have mapped the spatial variability of drug responses within biopsies and have tracked sample-to-sample variability. Sample heterogeneity observed in the biodynamic maps is associated with histological heterogeneity observed using inverted selective-plane illumination microscopy.

**Conclusion**: We have demonstrated the utility of TDSI as a functional imaging method to measure tumor heterogeneity and its potential for use in drug-response profiling.

## 1 Introduction

Tumor heterogeneity presents a challenge for the successful treatment of cancer using chemotherapeutics.^1^ For instance, genetic variability in tumors caused by clonal outgrowth of selected genotypes within a tumor may cause subsets of cells with genetic variations to be resistant even while the majority of the tumor responds to treatment. Selective pressure and genetic drift of the cancer cell population during treatment often lead to patient relapse and the emergence of broad chemoresistance and refractory disease.^2^^–^^4^ In addition to genetic heterogeneity, there is also spatial heterogeneity in tumor tissue arising from varying tissue constituents as well as varying microenvironments, including differences in extracellular matrix and connective tissues. The tumor microenvironment^5^^,^^6^ and epigenetic variations^5^^,^^7^^–^^9^ pose significant challenges to the selection of treatment based on genetic profiles. This has led, as an alternative, to phenotypic profiling^10^^–^^12^ of cancer tissue that captures the systemic response of cancer tissue to applied therapy. The challenge for phenotypic profiling of cancer tissue is the need to image intact microenvironments deep inside tissue, far from surface damage caused by surgical resection, and deep inside transport-limited regions that experience hypoxia, nutrient depletion, and metabolite build-up.

Optical coherence imaging (OCI)^13^^,^^14^ is a deep-tissue coherence-domain imaging approach based on digital holography^15^^–^^17^ that is a form of full-frame optical coherence tomography.^18^^,^^19^ Dynamic speckle in OCI images caused by dynamic light scattering from intracellular motions enables biodynamic imaging (BDI)^20^ to use intracellular dynamics as a unique form of image contrast. The changes in intracellular motions caused by applied therapeutics have been studied using tissue dynamics spectroscopy (TDS)^21^ to separate the effects of drugs across broad spectral bands and to capture specific signatures from different classes of drugs with different mechanisms of action.^22^ Preclinical trials of therapy responsivity assessment have been completed using TDS in spontaneous canine B-cell lymphoma and in ovarian xenografts.^23^^,^^24^ In a substantially different setting, assisted reproductive technology correlates the viability of cumulus-oocyte complexes with parameters from sample fluctuation power spectra.^25^

**Fig. 1:**
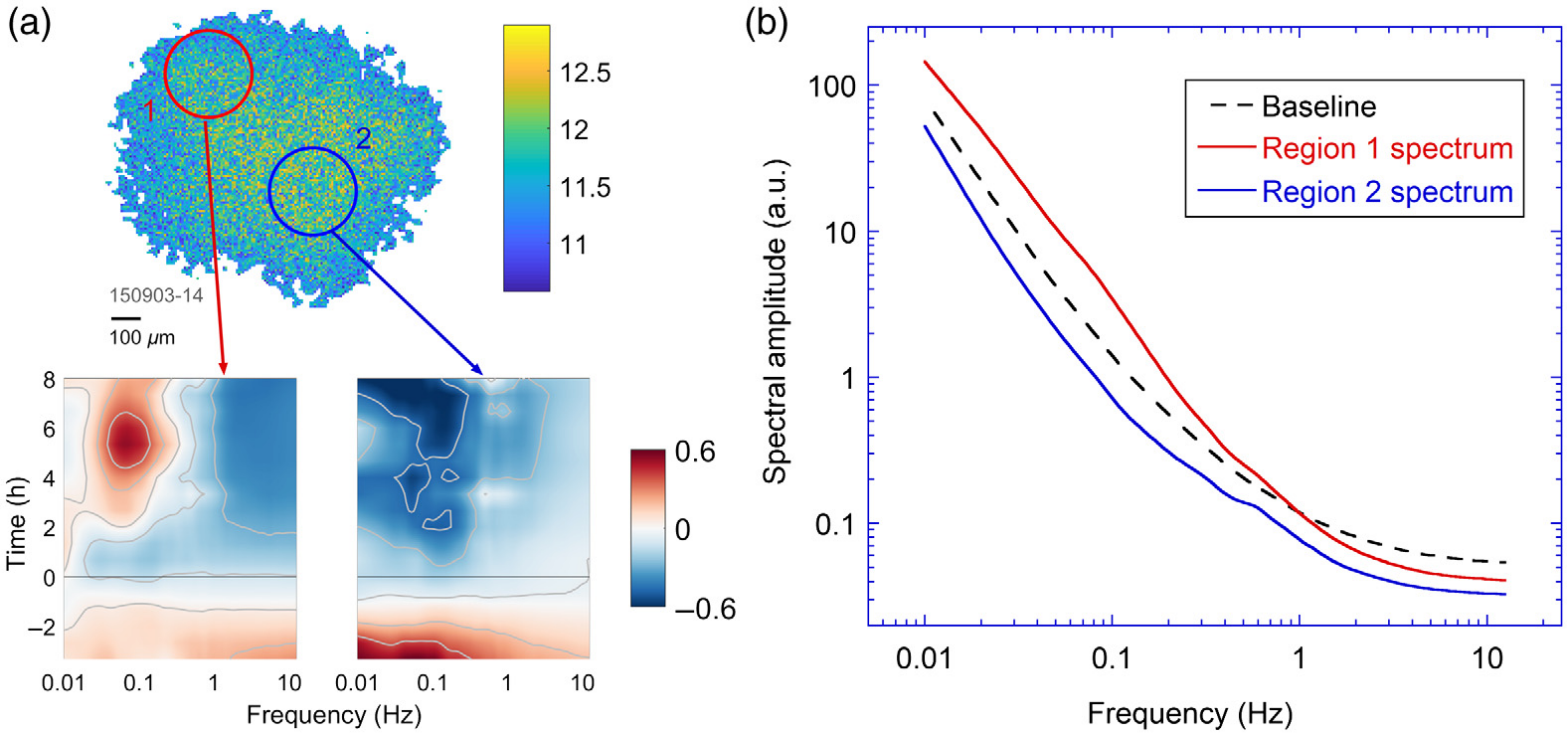
(a) An OCI image of a human esophageal biopsy and differential spectrograms (defined below) for the two circled areas. At time t=0 (black line), 100 μL of 25  μM cisplatin and 25  μM fluorouracil (5fu) combined solution is added to the sample. The two regions have significantly different responses: region 1 shows enhancement in low frequency and suppression in high frequency (redshift), while region 2 has suppression across all frequencies. (b) The terminal spectra of regions 1 and 2, respectively, compared to the average sample baseline spectrum.

The methodology of TDS is usually applied to entire samples that can be as large as $1\;{\mathrm{mm}}^{3}$ in volume (e.g., biopsies). However, intrasample variability in the TDS signatures poses a challenge for the prediction of patient response to therapy. While previous work using TDS has identified and characterized the different baseline conditions and drug responses in the “shell” and “core” areas of the samples,^21^^,^^22^^,^^25^ in that analysis, the boundary between the shell and core was arbitrarily defined. Some samples have a more complicated drug response structure than a simple “shell” and “core” model, as shown in Fig. 1, where the sample shows variation in both strength and pattern in its drug response in the two areas.

To address this problem, we introduce a functional imaging method called tissue dynamics spectroscopic imaging (TDSI) that evaluates sample response on a pixel level. In addition to a full-duration response map, the response can be segmented along the time axis to derive the time-lapse evolution of drug response, which can reveal the different rates at which a drug acts on each area. This methodology offers a quick, intuitive visualization of sample heterogeneity and drug effects. TDSI differs from dynamic-contrast OCT performed with off-axis holography,^26^ full-field OCT,^27^ or conventional OCT^28^^–^^33^ because its imaging contrast arises from shifts in intracellular dynamics caused by applied therapeutics rather than from baseline intracellular dynamics. In this way, TDSI is functional imaging that is specific to drug efficacy and displays the heterogeneous response of tissue to therapies.

## 2 Materials and Methods

### 2.1 Sample Preparation

Biological samples used in this paper include tumor spheroids grown from the DLD-1 intestinal adenocarcinoma cell line (ATTC, Manassas, Virginia) and human esophageal tumor biopsies. (IRB-approved IUCRO-0486 clinical trial. Written consent was obtained for each patient.) The multicellular DLD-1 spheroids mimic small avascular *in vivo* tumors by having an active growth zone on the outside of the spheroid and regions of apoptotic and necrotic cells progressively toward the core as nutrients and oxygen become rate-limiting for cell growth, but otherwise the spheroids are highly homogeneous. In contrast, the esophageal tumors are highly heterogeneous, with complex physiology and consist of different cell types. DLD-1 spheroids were grown in Corning U-bottom 96-well spheroid plates, and esophageal tissues were obtained by pinch biopsy from human patients. Tumor biopsies were collected and transported in chilled RPMI-1640 medium with HEPES buffer and cut into small pieces of 1 mm size or less. Both types of samples were immobilized in 1% low-gel-temperature agarose in the RPMI-1640 basal medium. Immobilized samples were overlaid with RPMI-1640 containing 10% heat-inactivated fetal calf serum (Atlanta Biologicals), penicillin (100 IU), and streptomycin (100  μg/mL).

Sample heterogeneity observed using TDSI was verified with high-resolution three-dimensional (3-D) images obtained from inverted selective plane illumination microscopy (iSPIM). To prepare samples for iSPIM imaging, 10% neutral buffered formalin was injected into each well to fix the tissues. After being washed with PBS, the samples were stained with 50-μM DRAQ5 (Biostatus, Ltd.) overnight with gentle agitation, then 0.5  mg/mL 80% ethanol-based Eosin Y (E4009, Sigma-Aldrich) for 4 h. Afterward, the agarose embedding the samples in the dish wells was removed, and samples were then washed with DI water three times, and PBS once followed by being immersed in X-CLARITY mounting solution (Logos Biosystems) for 15 min. Finally, the samples were fixed in the imaging chamber with silicone glue and immersed in X-CLARITY mounting solution for iSPIM imaging. After being imaged, the samples were processed for traditional H&E through the Tulane Medical School Histology Department. Four-micrometer-thick sections were cut and stained until each tissue was exhausted. The total processing time is about 16.5 h in total after fixation, including the PBS/di-water washing time. Imaging of each sample takes about 1 min average scanning time. Full-resolution image processing of each sample takes ∼50  min, including time for reconstruction, making pseudocolor images, and visualization.

### 2.2 Tissue Dynamics Spectroscopy

**Fig. 2:**
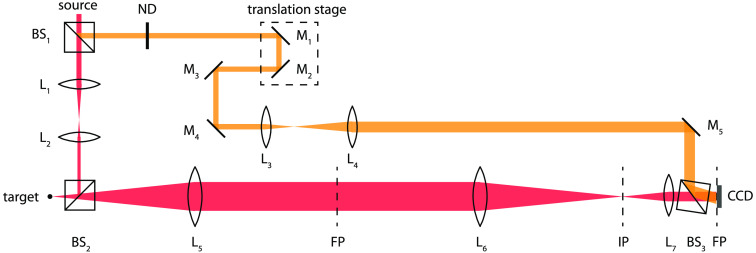
A schematic of the interferometer with a Mach–Zehnder off-axis digital holography configuration. The camera is on the Fourier plane. Optical sections are reconstructed using a 2-D spatial Fourier transform. ND, neutral density filter; $L_{1}-L_{7}$, lenses; ${\mathrm{BS}}_{1}-{\mathrm{BS}}_{3}$, beam splitters; $M_{1}-M_{5}$, mirrors; FP, Fourier plane; IP, image plane; $f_{5}=f_{6}=150\;\mathrm{mm}$; $f_{7}=50\;\mathrm{mm}$. The translation stage defines the coherence gate for time-domain ranging.

Speckle fluctuation dynamics of biological samples were measured and analyzed using a BDI system. The system optical configuration is a Mach–Zehnder interferometer, shown in Fig. 2. The light source is a Superlum S840-B-I-20 superluminescent diode with a center wavelength at 836.2 nm and a full power output of 22.9 mW with a bandwidth of 50 nm and a coherence length of ∼10  μm. A QImaging EMC2 camera is used for image acquisition. Lenses L5 and L6 constitute a 4f imaging system to an image plane (IP). The IP is subsequently transformed by lens L7 to the Fourier plane located at the CCD pixel array. A single sample image is obtained by performing a two-dimensional (2-D) FFT on the hologram captured by the CCD camera located on the Fourier domain, and one of the two conjugate images is stored as a 256×256 array.^21^ Because of the use of long focal lengths ($f_{5}=f_{6}=150\;\mathrm{mm}$), the speckle size on the camera plane is ∼60  μm, and the reconstructed object-plane point-spread function is ∼15  μm spanned by three pixels.

Two data acquisition formats are used for data presented in this paper: a format containing 2048 frames captured at 25 fps, and a format with 500 frames at 25 fps and 50 frames at 0.5 fps. The second format has a smaller data storage requirement but requires a stitching algorithm to create a single spectrum.^34^ Within this paper, only DLD samples use the 2048 frame format, while the esophageal samples used the 500/50 frame format to assure comparability among spectra within a given study. A typical experiment has six time-frames of baseline measurements and 15 time-frames of drug response measurements looping repeatedly through 16 wells in a 96-well plate format. A single loop through all 16 samples takes about 40 min.

The fluctuation power spectrum from one pixel at position (i,j) in the sample is calculated as the square of the FFT of the intensity time series:

$$S(i,j;f)={\left|F_{t}[I(i,j;t)]\right|}^{2}.$$ (1)

The average spectrum of a region σ is calculated as

$$S_{\sigma ,\text{raw}}(f)={\left\langle S(i,j;f)\right\rangle }_{i,j}=\frac{\underset{(i,j)\in \sigma }{\sum }S(i,j;f)}{\underset{(i,j)\in \sigma }{\sum }1},$$ (2)

where σ is a spatial mask segmenting the entire sample. The “raw” spectrum is normalized based on Parseval’s theorem:^34^

$$S_{\sigma ,\text{norm}}(f)=\frac{S_{\sigma ,\text{raw}}(f)}{\underset{f}{\sum }S_{\sigma ,\text{raw}}(f)},$$ (3)

i.e., dividing the raw spectrum by the sum of all frequency components (including the DC component). The full-length spectrogram of segment σ is a time-lapse sequence of differential spectra where each spectrum at time τ is calculated as a normalized logged spectrum subtracted by the baseline spectrum averaged over the full sample:

$$dS_{\sigma }(f;\tau )=\log\;S_{\sigma ,\text{norm}}(f;\tau )-\log\;\frac{1}{N}\overset{N}{\underset{i=1}{\sum }}S_{\sigma ,\text{norm}}(f;\tau =\tau_{i}),$$ (4)

where N is the number of baseline loops.

### 2.3 Biodynamic Biomarkers and TDS Visualization

Biomarkers that evaluate sample preconditions, also called baseline conditions, before a treatment is applied include backscatter brightness, normalized standard deviation, spectrum knee frequency, and spectral slope (S).^32^ Biomarkers that evaluate drug responses include changes in these biomarkers after application of drugs as well as features extracted from spectrograms. This paper focuses mainly on the drug response and features from spectrograms.

**Fig. 3:**
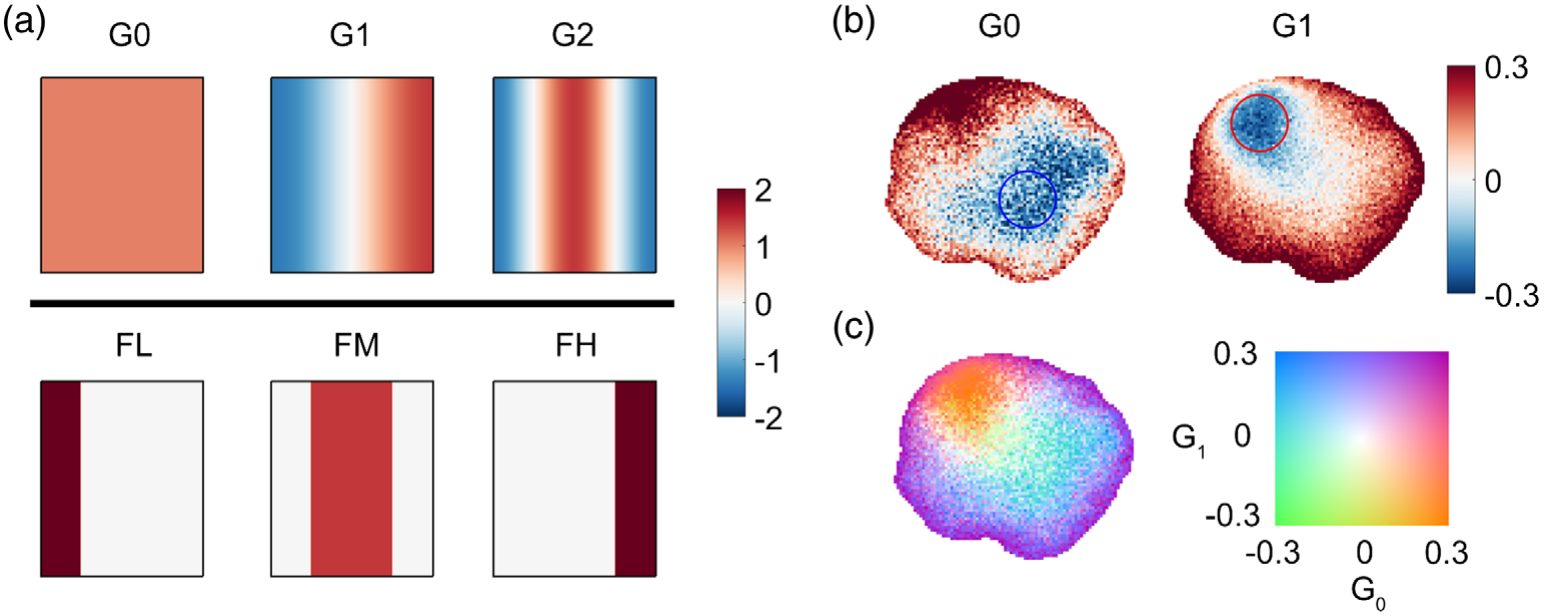
(a) A subset of the spectrogram masks used in the color merge. (b) Two maps of drug response of an esophageal sample “150903-14” (same as in Fig. 1) exposed to cisplatin and fluorouracil combination therapy under masks G0 and G1. (c) A “merged” bivariate color image with its 2-D color map.

Despite the differences in drug responses related to sample baseline conditions and drug mechanisms, a biodynamic drug spectrogram usually has one of a limited number of patterns. Spectroscopic masks (time-frequency filters) are designed to match the characteristics of the spectrograms, a few of which are shown in Fig. 3(a). The top three patterns G0, G1, and G2 form a set of “orthonormal” masks that are related to the broadband (in the sense of frequency components) pattern of a spectrogram, while the bottom three patterns FL, FM, and FH form another set of masks related to local response patterns. These frequency bands, and their associated frequency cutoffs, can be related to changes in intracellular motions and their related speeds.^23^^,^^35^ Although the individual biomarker values depend on the choice of cut-off frequencies, the orthonormal character of the masks provides a unique representation of the spectrogram, and this representation is used in pattern recognition algorithms. For each mask, a feature value is obtained by calculating the inner products of the spectrogram and the mask, i.e., projecting the spectrogram onto the mask,^22^ and the features of a spectrogram are represented by a vector of feature values.

After condensed-data-format files are generated (refer to Sec. S.1 in the Supplemental Material), a differential spectrogram for each TDSI pixel (referred to as a “microspectrogram”) is calculated as

$$dS_{\sigma (\overset{˜}{i},\overset{˜}{j})}(f;\tau )=\log\;S_{\sigma (\overset{˜}{i},\overset{˜}{j}),\text{norm}}(f;\tau )-\log\;\frac{1}{N}\overset{N}{\underset{i=1}{\sum }}S_{\Sigma ,\text{norm}}(f;\tau =\tau_{i}),$$ (5)

where the average baseline spectrum of the entire sample is used instead of the TDSI pixel’s “own” baseline. For a given mask, a feature value (discussed above) can be calculated for each pixel, and the feature values of an entire sample produce a 2-D image called a TDSI. Two TDS images under the “G0” mask and “G1” mask (blueshift associated with increased intracellular speeds) are shown in Fig. 3(b). For the “G0” TDS image, the area in the blue circle has negative values, indicating broadband decrease in activity, which matches the differential spectrogram of the circled area of the same sample as shown in Fig 1(a). Similarly, the spectral response in the red circle area agrees with the positive values in the same area in the “G1” TDS image.

As discussed above, the sample in Fig. 3 has a large variation in drug response in terms of strength and spectral patterns, displayed by the distribution of values in the TDSI of Fig. 3(b). Both “G0” and “G1” images show a change in magnitude and sign from bottom left to top right (corresponding to changes in the strength of drug response, referred to as “intramask heterogeneity”), and the “G0” image has a different pattern than “G1,” where “G0” has strong negative values on the bottom right while “G1” has strong negative values in the upper left (corresponding to changes in pattern, referred to as “intermask heterogeneity”). To better visualize the variation, bivariate color images are introduced to produce a single visualization that captures drug responses across the entire sample, where each “variable” is a feature value of a mask. Feature values from two masks are a convenient way to illustrate drug-response heterogeneity within a sample, and the following discussion will focus on bivariate representations of drug response.

Bivariate color maps are used in cartography^36^^,^^37^ and medical imaging,^38^ and many studies have addressed how to choose proper colormaps for bivariate data visualization.^39^^–^^41^ We have elected to use the “Teuling3” colormap in the following visualizations, which is generated by linearly interpolating four colors at the four corners in the sRGB space plus a “whitening” core in the center.^39^^,^^42^ This color map has good color saturation, relatively equal visual impact, and a zero value appears as white, which is consistent with the diverging “blue-red” one-dimensional colormap used in our spectrograms and univariate TDS maps. Figure 3(c) shows a bivariate image of a human esophageal biopsy sample “merged” from the two univariate TDS maps in Fig. 3(b).

When a sample has areas with spectrograms that are the inverse of each other, they cancel each other out in an average over the full sample, resulting in a mild spectrogram and near-zero feature values. In this case, the weak average response belies the strong change in the intracellular dynamics and the fluctuation spectra, and this can lead to the misinterpretation that the sample does not respond to the drug. To address this problem, two new biomarkers that evaluate sample heterogeneity are added to the “traditional” average spectrogram-based biomarkers. The two biomarkers evaluate the “intramask” and “intermask” heterogeneity, respectively. To achieve a high signal-to-noise ratio, TDS images are first (re)generated with an 8×8  pixel averaging (instead of the standard 2×2  px). The coarse eight-pixel averaging is used only to calculate the heterogeneity biomarker values of a sample at a scale of 40  μm and above. Finer scale is not necessarily meaningful for quantifying spatial heterogeneity across a millimeter sample. The feature values are bounded to a range $\left[-A_{\mathrm{th}},A_{\mathrm{th}}\right]$ and then assigned “scores” ranging from 0 to 1 for both heterogeneity evaluations, calculated using the following steps:

1. Select a set of n masks
2. For each mask u, calculate $\Delta a^{u}$ and $\Delta [\mathrm{sgn}(a^{u})]$
3. For each mask pair u-v, calculate $\left|\rho (a^{u},a^{v})\right|$ and $\left|\rho [\mathrm{sgn}(a^{u}),\mathrm{sgn}(a^{v})]\right|$
4. The first biomarker denoting overall intramask heterogeneity is calculated as
  $$h_{1}=\frac{1}{2n}[m_{1}\overset{n}{\underset{i=1}{\sum }}\Delta a^{u}+m_{2}\overset{n}{\underset{i=1}{\sum }}\Delta \mathrm{sgn}(a^{u})].$$ (6)
5. And the second biomarker representing overall intermask heterogeneity is calculated as
  $$h_{2}=1-\frac{1}{n(n-1)}\underset{i-j\text{ pairs}}{\sum }\{|\rho (a^{u},a^{v})|+|\rho [\mathrm{sgn}(a^{u}),\mathrm{sgn}(a^{v})]|\},$$ (7)
  where $a^{u}=\{a^{u}(\tilde{i},\tilde{j})\}$ are values of the TDS image of mask u, $\mathrm{sgn}(a^{u})$ is a map of signs of $a^{u}$, $\Delta a^{u}$ is the standard deviation of $a^{u}$, $\rho (a^{u},a^{v})$ is the correlation coefficient of $a^{u}$ and $a^{v}$, and $m_{1}$ and $m_{2}$ are normalization factors
  $$\left\{ \begin{array}{l} m_{1}=\frac{1}{A_{th}}\\ m_{2}=1 \end{array},\right.$$ (8)
  based on Popoviciu’s inequality on variances.^43^ We use $A_{\mathrm{th}}=0.3$ and n=3, and the local masks FL, FM, and FH are used for heterogeneity scores.

Based on these definitions, the extreme values are achieved under these cases: $h_{1}=0$ when n TDS images are completely uniform (totally homogeneous), and $h_{1}=1$ when TDS images have only two values of opposite signs in an equal number of pixels. When the TDS images are completely nonlinearly correlated to each other, then $h_{2}=0$. In the opposite case when all values are completely correlated then $h_{2}=1$. Examples will be provided in the next section to illustrate these two heterogeneity benchmarks.

### 2.4 Time-Lapse Drug Response Visualization

After a drug is added to a biopsy sample, the change in its intracellular dynamics is usually not immediate and depends on the drug mechanism of action, especially for drugs targeting DNA that produce slow cellular responses. Also, some parts of the sample may respond to a drug faster than the entire sample. TDSI allows us to study both the time delay and nonuniformity in drug action, which is called time-lapse TDSI. Instead of extracting feature values from full-length spectrograms, time-lapse TDSI uses responses within a small moving time “window” of the spectrogram. Examples are included in the following sections.

### 2.5 Inverted Selective Plane Illumination Microscopy

The iSPIM system is constructed around a commercially available diSPIM platform (ASI) and has been described in previous publications.^44^^,^^45^ In brief, two immersion objectives (CTO, ASI/Special Optics, 15.3X−17.9X) are orthogonally mounted above the sample, with each at a 45-deg angle from the norm, enabling traditional sample preparation. Dual-view imaging is possible by alternating roles of the two objectives as illumination and detection, but only single-view was adopted for this paper. Volumetric images were obtained by moving the sample with respect to the objectives. To cover the whole area of the sample, multiple y strips were acquired with about 20% overlap between two adjacent strips. After imaging was completed, the images were first shifted and interpolated with custom MATLAB code to recover its 45-deg angle.^46^ Multiple paths were then stitched with Fiji plugin.^47^ The DRAQ5 and eosin images (D&E) were remapped to a composite RGB stack to simulate the traditional H&E colors.^48^ Finally, 3-D reconstruction of the D&E images was obtained from the alpha blending mode of 3-D viewer of Vaa3D.^49^^,^^50^ The mounting media is Xclarity with a refractive index of 1.45, that when combined with the NA of 0.4, gives a magnification M of 16.7× with a lateral resolution of 0.76  μm and an axial resolution of 3.8  μm.

## 3 TDSI Results

**Fig. 4:**
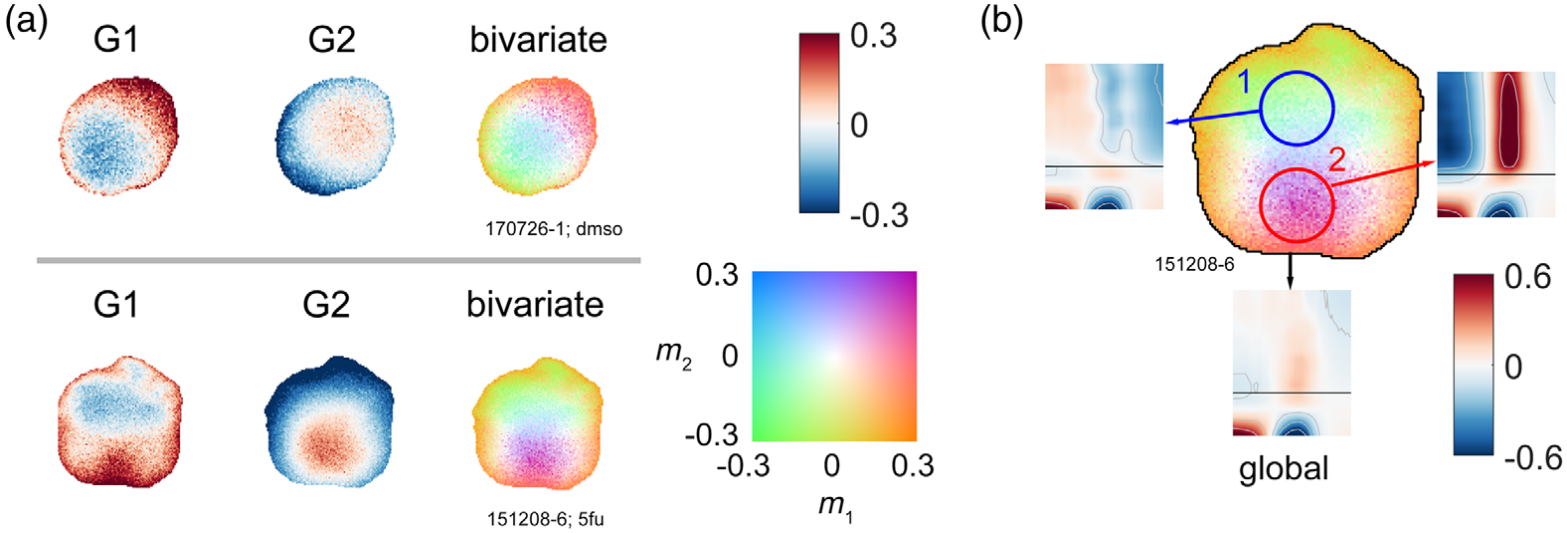
(a) Bivariate color representation of drug responses of two samples treated with different drugs, showing two univariate maps and a “merged” bivariate color map. The first sample was refreshed with DMSO, while the second sample was treated with 25  μM fluorouracil (5fu). (b) Global and regional spectrograms of sample “151208-6” from (a). The global spectrogram has a relatively weak response (max<10%), while the two circled areas have 30% to 60% enhancement or suppression. Drugs were added at t=0 (black line on spectrograms).

A large number of esophageal biopsies display spatially heterogeneous responses to drugs. In Fig. 4(a), two biopsy samples that have large intramask heterogeneity are presented in univariate and bivariate forms. Sample “151208-6” has a weak response when averaged over the whole-sample spectrogram [Fig. 4(b) “global”], because the local areas “1” and “2” have strong but opposite responses that tend to cancel in the sample average.

**Fig. 5:**
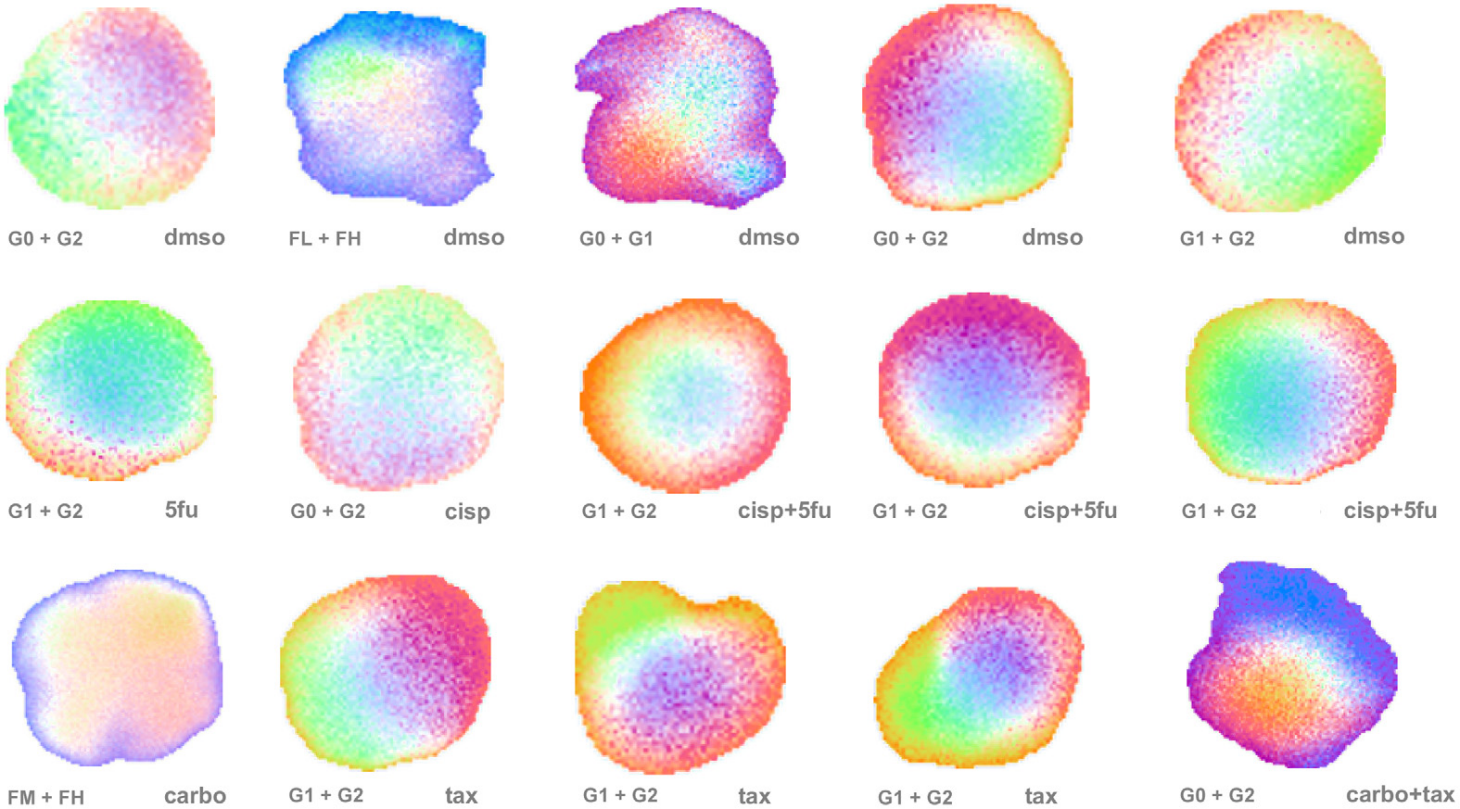
More examples of bivariate TDS images showing sample-to-sample variability in drug responses. Masks are designated in the lower left corners of images, while lower right corners designate drug treatments. Drug abbreviations: DMSO, 0.1% DMSO in growth medium (used as a negative control); cisp, 25  μM cisplatin; 5fu: 25  μM fluorouracil; tax, 5  μM paclitaxel; carbo, 25  μM carboplatin. “+” indicates a combination of two drugs. The color map and scale are the same as in Fig. 3.

An assortment of bivariate TDS images is shown in Fig. 5. Some samples have relatively uniform color in the “merged” map, indicating smaller variation in the biomarker values, while others have a rainbow-like smooth transition across the sample, which is related to high heterogeneity in the drug response.

**Fig. 6:**
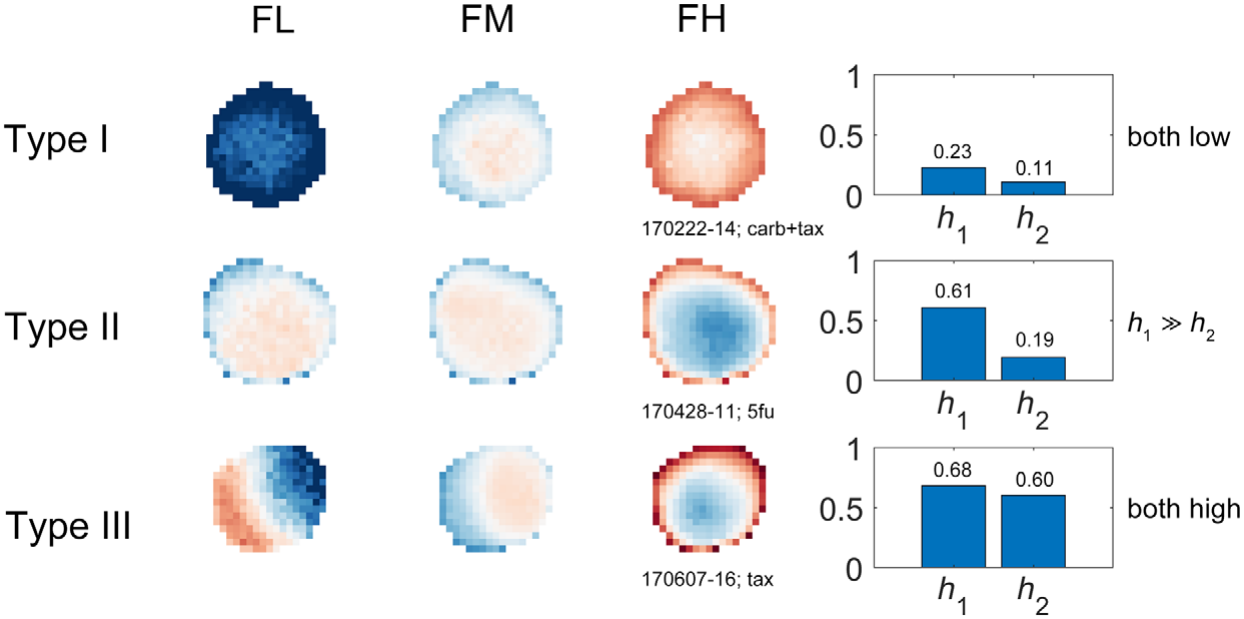
Three types of samples that have different levels of same-mask heterogeneity and cross-mask heterogeneity, with scores on the right.

There are roughly three types of heterogeneity, shown in Fig. 6 along with their $h_{1}$ and $h_{2}$ scores: (i) Type I are samples that have almost uniform responses under all masks. (ii) Type II samples have spatial variability but show similar patterns across different masks. (iii) Type III samples have TDS images with nonoverlapping strongly responding areas. Types I and III are the most common.

**Fig. 7:**
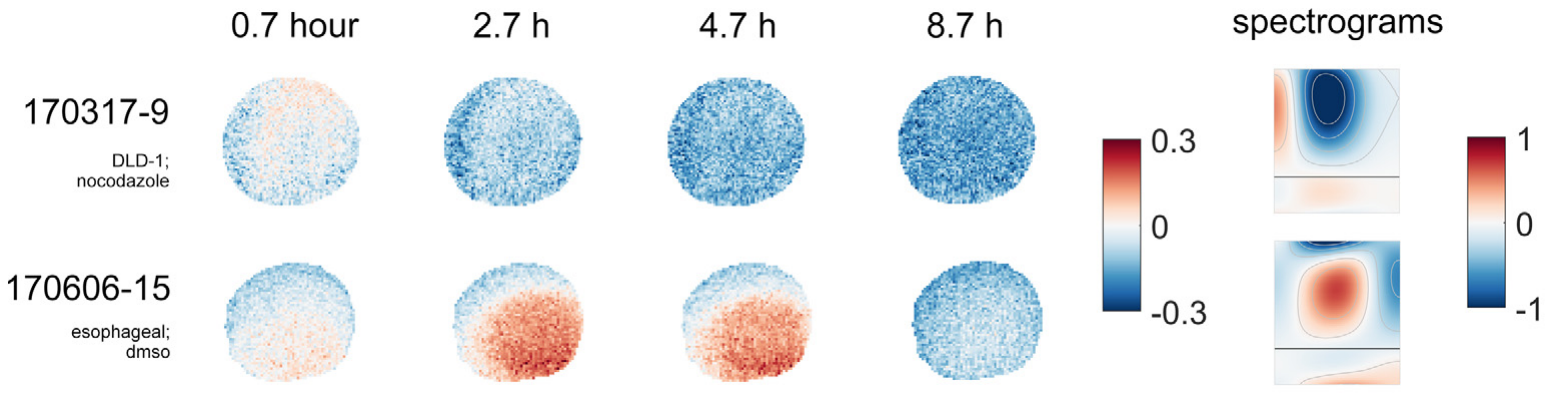
Time-lapse TDS image of samples responding to drugs. Sample “170317-9”: response of a DLD spheroid sample treated with 10  μM nocodazole [same as in Fig. 3(d)] with the G2 mask, showing a silent core shortly after drug was added (0.7 h), which was “invaded” by the drug and later achieved a spatially homogeneous response (2.7 to 8.7 h). Sample “170606-15”: response of an esophageal biopsy sample in the control medium, also under the G2 mask.

Time-lapse images offer an additional layer of understanding of the spatial evolution of drug effects or sample conditions. In Fig. 7 for sample “170317-9” treated with nocodazole, the blue response pattern (mid-frequency suppression and low- and high-frequency enhancement) grows stronger over time before saturation, which indicates that nocodazole’s suppression of microtubule polymerization begins at the outer periphery and slowly penetrates the core of the sample. As another example, the red area in the TDS image of sample “170606-15” becomes stronger until around 9 h, when the sample displays an overall suppression across the entire sample.

## 4 Comparison with Inverted Selective-Plane Illumination Microscopy

Given that BDI is a 3-D imaging technology that uses low-coherence light, the different drug response phenotypes revealed by TDSI may be related to different types of tissues in a certain region of a sample. A complementary 3-D imaging technique is iSPIM that produces microscopic images of 3-D slices with high lateral and axial resolutions, which allows us to distinguish features in the images. Therefore, by comparing TDSI with iSPIM, we can investigate whether the heterogeneity related to drug response variability from TDSI is also present in microscopic images, i.e., link dynamic information from functional imaging with the histology of biological tissues.

**Fig. 8:**
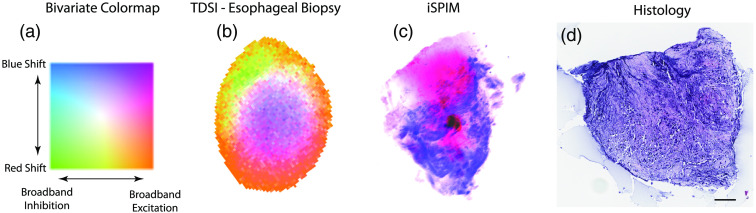
TDSI map, iSPIM 3-D-reconstructed image and H&E histology image for an esophageal biopsy sample. All scale bars are 100  μm. (a) Bivariate colormap representing G0 (broadband inhibition) and G1 (blue shift). (b) Bivariate TDSI map of human esophageal biopsy responding to 5-fluorouracil (5-fu) with G0 and G2 as the two masks. The color map and color scale are the same as in Fig. 3. (c) The iSPIM image with DRAQ 5 (blue) and Eosin (pink) for the same sample in (a), and (d) histology image. The orientations of the images are not registered.

As an example, TDSI maps for sample 190801-15 are compared against its iSPIM images and H&E histology images in Fig. 8. This sample is a pinch biopsy from a patient who was resistant to neoadjuvant therapy. The biodynamic response is mapped for the tissue response to 5-fluorouracil. In the TDSI map, there is a distinct central region (purple) contrasted with the outer regions (orange). In the bivariate colormap, purple represents broadband excitation with a blue shift, indicating an activated response of the tissue to the 5-fu. The central region is likely to be naturally hypoxic, which can affect the mechanism of the drug. In a related study of esophageal cancer patients, a blueshift induced by 5-fu is representative of a beneficial response to the chemotherapy. In contrast, in the peripheral tissue, that is hyperoxic, the broadband excitation is accompanied by a redshift. Furthermore, the green region indicates overall suppressed activity (broad inhibition accompanied with a redshift of slower intracellular speeds). In comparison to the TDSI, in the iSPIM image the lower part matches the lower part of the histology image containing a large concentration of DNA. The upper part of the iSPIM image is cytoplasm or unstained tissues, matching the lack of nuclei in the upper part of the histology image, which potentially indicates collagenous connective tissues. Although the image orientations were not registered between the techniques, the images in Fig. 8 demonstrate spatial polarity in the tissue types. Future studies to compare TDSI with iSPIM would register sample orientation to identify TDS spectral signatures of connective tissue relative to epithelial tissue. The work presented here is proof-of-principle that spatial heterogeneity can be observed in both imaging modalities.

In this comparison, the advantage of iSPIM is the ability to acquire microscopic image with high resolution and detailed cellular-level structural information with acceptable speed. This is in contrast to TDSI which achieves only tissue-scale imaging resolution (∼15  μm voxel size). On the other hand, TDSI is a functional imaging method that is highly specific to the action of drugs on tissue dynamics, while iSPIM can only provide structural information without being specific to drug response. Furthermore, TDSI is nondestructive, allowing longitudinal studies that can span several days, including the ability to apply drugs and to clear them. Both techniques have the advantage of imaging into 3-D tissues, which is a key aspect of maintaining the tumor microenvironment during imaging.

## 5 Discussion

BDI is a tool that is sensitive to intracellular dynamics and has been applied successfully to phenotypic profiling to predict patient outcomes. For instance, sample motility and dynamic biomarkers have been shown to be consistent and reliable indicators of pharmacodynamics effects. However, these biomarkers are usually calculated as whole-sample averages when used in classification and similarity analyses, overlooking intrasample heterogeneity. The introduction of TDSI solves this problem by evaluating the responses of subregions of the sample to reveal new information on the complex spatial structures in sample drug response, which is supported by evidence from other imaging techniques such as iSPIM and histology. Visualization of sample heterogeneity is facilitated with a bivariate color representation and is quantitatively characterized by $h_{1}$ and $h_{2}$ scores. This imaging method is further extended to generate whole-sample time-lapse TDSI maps, providing a method to monitor drug mechanisms.

In addition to visualizing sample heterogeneity, TDSI maps can provide additional information and improve classification accuracy when evaluating anti-cancer drug effectiveness on a patient level. For samples with regions of opposite responses, the whole-sample average spectrogram may suggest a mild response to the drug, making the sample and the patient appear to be less sensitive to the treatment. The proposed solution here is to introduce additional biomarkers that characterize regional drug responses. As an example, the sample shown in Fig. 3(b) can be split into two regions based on the sign of G1 biomarker values (which can be related to the sample’s heterogeneous structure), and the feature values of these two regions can be calculated. The set of feature values that capture both sample average response as well as regional response would provide a more comprehensive assessment of the patient.

TDSI can be extended for further imaging and analysis applications. Since BDI is a 3-D imaging technique and achieves depth selection with coherence gating, a volumetric TDS image can be generated by scanning different slices of a sample. Also, time-lapse TDS analysis is a quantitative approach to visualize drug-action time dependence. Features such as delay and distribution related to pharmacokinetics and pharmacodynamics (PK/PD) could be obtained from time-lapse images to provide insight into processes such as dose-response relationships.

Challenges to TDSI include sample immobilization and multiple light scattering. TDSI evaluates the drug spectral response on the pixel level and requires that the same part of the sample is imaged throughout the experiment. This requires the sample to maintain the same lateral and axial positions. In addition, multiple light scattering induces aberrations of the image and reduces the signal-to-noise ratio, which makes TDSI more effective at shallower depths.

TDSI is an important extension to the current suite of BDI modalities (OCI, MCI, and TDS). MCI maps are simple and intuitive functional images that visualize sample motility and have revealed the contrast between a viable shell and a necrotic core for rat tumor spheroids.^26^ TDSI, by comparison, generates a set of more detailed functional maps that complement MCI because the critical frequencies in spectral masks used in TDSI are related to specific types of intracellular components and motions, offering a comprehensive view of changes occurring in the sample. TDSI is a versatile functional imaging method that could provide new information for drug-response profiling and has the potential for improving predictions of response to therapy and drug screening.
